# Misclassifying and differentiating metacognitive therapy: conceptual and methodological issues in Stenzel et al.

**DOI:** 10.1017/S0033291725101463

**Published:** 2025-08-15

**Authors:** Robin Bailey

**Affiliations:** The School of Psychology, University of Greater Manchester, Bolton, UK

Dear **Editor-in-Chief**,

I read with interest the recent article by Stenzel et al. (2025), titled *‘Efficacy of cognitive behavioral therapy in treating repetitive negative thinking, rumination, and worry: a transdiagnostic meta-analysis’.* The authors provide a valuable and methodologically ambitious review of CBT-based interventions for repetitive negative thinking (RNT). I commend the scope of the project, particularly its transdiagnostic framing and effort to differentiate treatment effects across therapeutic subtypes. However, several critical aspects require clarification.

First, the article states that Metacognitive Therapy (MCT) was ‘not explicitly designed to address RNT according to the current frameworks (Ehring & Watkins, 2008)’. This misrepresents both the historical and theoretical intent of MCT and the cited framework itself. Developed by Wells (1995, 2009) based on the model of Wells and Matthews (1994), MCT was the first therapy specifically formulated to target worry and rumination, mechanisms widely recognized as central to RNT. Recent historical analyses describe MCT as a theory-driven intervention that systematically conceptualizes and treats the core processes of RNT, representing a major advance in psychotherapy (Capobianco & Nordahl, 2023). Ehring and Watkins (2008) explicitly identify Wells’ metacognitive model as foundational to the RNT construct, noting that worry and rumination are ‘*repetitive, difficult to control and negative in content*, and possibly related to positive and negative meta-cognitions’ (p. 200). These features are adopted by the review itself as definitional. To suggest that MCT does not explicitly address RNT is therefore inaccurate and conceptually incompatible with both the cited framework and the review’s operational definition.

Second, although the authors preregistered their intention to compare ‘CBT that specifically addresses RNT’ versus ‘general approaches’, the method of classification was not defined in the PROSPERO protocol (CRD42023453038) or the OSF preregistration (DOI: 10.17605/OSF.IO/WDBFJ). The registered eligibility criteria included interventions based on CBT models (explicitly listing ‘MCT, ACT, and DBT’) but did not specify a coding rule requiring that studies include an explicit textual statement that at least ‘one intervention’ targeted RNT. The rule applied in the published review, grouping studies as ‘specific’ only if they self-identified as targeting RNT, appeared to be introduced post hoc. No justification or protocol amendment was provided. This constitutes a material deviation from the preregistered plan. Given that one of the review’s central conclusions relies on this subgroup distinction, the absence of preregistered criteria raises significant concerns about analytic transparency and interpretive robustness.

Third, the specific subgroup coding rule described in the article is: ‘Treatment arms were coded as “specific” if the study explicitly stated that at *least one intervention addressed RNT, worry, or rumination referring to the relevant concept.* Otherwise, it was coded as “general”’. This lexical criterion, requiring explicit mention of targeting RNT or its components, was applied inconsistently. For example, Wells and Colbear (2012) describe helping patients ‘discriminate between an intrusive thought and memory and subsequent extended processing in the form of worry and rumination’, followed by a ‘worry/rumination postponement experiment’: an established worry intervention. Similarly, Nordahl et al. (2018) report that ‘In MCT the goal is the introduction of an alternative set of strategies so that the patient is better able to regulate worry’, concluding that ‘MCT had a better outcome in reducing worry at post-treatment’. Both studies clearly refer to RNT-relevant concepts and describe interventions designed to address them, satisfying the stated criteria. Yet both were classified as ‘general’. No coding manual, inter-rater reliability indices, or decision rules for subgroup classification were reported. The result is a classification system that prioritizes surface phrasing over therapeutic mechanisms and appears inconsistently enforced, undermining the reliability of subgroup findings.

Fourth, in the discussion, the authors argue that they ‘did not include MCT as RNT-focused treatments by default’, on the grounds that MCT ‘seeks to change meta-cognitions potentially contributing to RNT’. This rationale is conceptually flawed. By the review’s own definition, RNT consists of ‘worry, rumination, and content-independent negative thinking’, all which MCT was developed to address directly. The core mechanism targeted by MCT the ‘Cognitive Attentional Syndrome’, is not tangential to RNT but constitutive of it. Whether a therapy states that it ‘targets worry/rumination’ or ‘targets the drivers of worry/rumination’, (MCT does both), the clinical aim is the same: to disrupt RNT. The justification offered rests on a semantic distinction between process and content that lacks theoretical substance. Consistent application of the review’s coding rule would have actually led to MCT’s inclusion as an RNT-specific intervention.

These classification decisions carry substantive implications. The review concludes that ‘RNT-specific’ CBTs (*g* = 0.99) are substantially more effective than ‘general’ CBTs (g = 0.56). However, if MCT, one of the most explicitly RNT-focused therapies, was misclassified and its effects allocated to the ‘general’ category, this distinction becomes unreliable. Multiple independent meta-analyses (e.g. Andersson et al., 2025) have shown that MCT frequently outperforms standard CBT, with between-group effect sizes ranging from *g* = 0.43 to *g* = 0.97. Misclassification of MCT risks distorting effect size estimates, misleading clinical interpretation, and compromising the review’s central claim.

I encourage future reviews to base subgroup classifications on theoretically grounded mechanisms of change rather than surface textual criteria. Full transparency regarding deviations from preregistered protocols is essential to safeguard analytic rigor. Accurately representing therapies such as MCT, whose rationale and techniques are explicitly RNT-focused, is critical for fair synthesis, effective clinical translation and advancement, and maintaining the integrity of the evidence base.

Yours Sincerely

**Robin Bailey**
